# Molecular Characterization of Multidrug Resistant Hospital Isolates Using the Antimicrobial Resistance Determinant Microarray

**DOI:** 10.1371/journal.pone.0069507

**Published:** 2013-07-25

**Authors:** Tomasz A. Leski, Gary J. Vora, Brian R. Barrows, Guillermo Pimentel, Brent L. House, Matilda Nicklasson, Momtaz Wasfy, Mohamed Abdel-Maksoud, Chris Rowe Taitt

**Affiliations:** 1 Center for Bio/Molecular Science and Engineering, US Naval Research Laboratory, Washington, D.C., United States of America; 2 National Research Council Postdoctoral Associate at US Naval Research Laboratory, Washington, D.C., United States of America; 3 US Naval Medical Research Unit No. 3, Cairo, Egypt; University of Malaya, Malaysia

## Abstract

Molecular methods that enable the detection of antimicrobial resistance determinants are critical surveillance tools that are necessary to aid in curbing the spread of antibiotic resistance. In this study, we describe the use of the Antimicrobial Resistance Determinant Microarray (ARDM) that targets 239 unique genes that confer resistance to 12 classes of antimicrobial compounds, quaternary amines and streptothricin for the determination of multidrug resistance (MDR) gene profiles. Fourteen reference MDR strains, which either were genome, sequenced or possessed well characterized drug resistance profiles were used to optimize detection algorithms and threshold criteria to ensure the microarray's effectiveness for unbiased characterization of antimicrobial resistance determinants in MDR strains. The subsequent testing of *Acinetobacter baumannii*, *Escherichia coli* and *Klebsiella pneumoniae* hospital isolates revealed the presence of several antibiotic resistance genes [e.g. belonging to TEM, SHV, OXA and CTX-M classes (and OXA and CTX-M subfamilies) of β-lactamases] and their assemblages which were confirmed by PCR and DNA sequence analysis. When combined with results from the reference strains, ∼25% of the ARDM content was confirmed as effective for representing allelic content from both Gram-positive and –negative species. Taken together, the ARDM identified MDR assemblages containing six to 18 unique resistance genes in each strain tested, demonstrating its utility as a powerful tool for molecular epidemiological investigations of antimicrobial resistance in clinically relevant bacterial pathogens.

## Introduction

The evolution, increasing prevalence and dissemination of pathogenic bacteria resistant to multiple antimicrobial agents is currently recognized as one of the most important problems in global public health [1]. The rapid spread of antibiotic resistance genes, facilitated by mobile genetic elements such as plasmids and transposons, has led to the emergence of multidrug resistant (MDR) strains of many clinically important species that now frequently leave clinicians out of therapeutic options [2], [3].

Traditional phenotypic methods currently used to determine antimicrobial resistance profiles (e.g., disk diffusion, broth microdilution) remain critical in guiding appropriate treatment options. However, techniques such as these are unable to determine the actual molecular mechanisms of resistance, and are especially lacking in situations where the observed phenotype is a result of the interaction of multiple gene products with overlapping activities [4]. Molecular techniques, such as PCR and DNA sequencing, have recently been employed to mitigate some of these deficiencies by identifying genes and genetic assemblages responsible for antibiotic resistance and MDR, monitoring the spread of resistance determinants, and elucidating the genetic elements responsible for the dissemination of resistance determinants.

The use of DNA microarrays is another promising technology for the identification of antimicrobial resistance determinants in any number of species. Due to their inability to determine whether resistance determinants are expressed or gene products are functional, DNA microarrays are not intended to replace standard phenotypic testing. Rather, microarrays provide a powerful platform for molecular epidemiology and broad-based screening. This contention has been supported by a number of recent reports that have successfully applied a variety of microarray platforms for both limited [5], [6], [7], [8] and broad spectrum [9], [10], [11] detection of antibiotic resistance genes. Furthermore, as they allow for the simultaneous detection of a large number of genes in a single assay, microarrays can be used to track determinants directed against multiple classes of antibiotics that are frequently found clustered in mobile genetic elements [12]. The rapid spread of such assemblages not only makes the successful treatment of infections increasingly difficult but also complicates epidemiological investigations aimed at elucidating the spread of antimicrobial resistance genes and the underlying genetic structures that produce MDR phenotypes.

In this study, we describe the Antimicrobial Resistance Determinant Microarray (ARDM), an electrochemically interrogated DNA microarray platform containing 2,241 oligonucleotide probes targeting 239 genes that confer resistance to 12 classes of antimicrobial compounds, quaternary ammonium compounds and streptothricin, and its accompanying sample processing and analysis methods that were developed for broad-range MDR gene detection. Using well-characterized and/or sequenced reference strains, we confirmed ∼25% of the ARDM content and successfully tested *Acinetobacter baumannii*, *Escherichia coli* and *Klebsiella pneumoniae* hospital isolates, demonstrating the applicability of this technology for the detection of antibiotic resistance gene assemblages in clinically relevant bacterial pathogens.

## Materials and Methods

### Bacterial strains

Genomic DNA preparations from reference strains of *A. baumannii*, *E. coli*, *K. pneumoniae*, *Staphylococcus aureus*, *S. epidermidis* and *Enterococcus faecalis* were purchased from American Type Culture Collection (ATCC), Manassas, VA (Table 1). Four *A. baumannii* clinical strains with published sequences [13] were obtained from the US Department of Defense Multidrug Resistance Surveillance Network (MRSN, Walter Reed Army Institute of Research, Bethesda, MD). Fifteen additional clinical strains with high levels of β-lactam resistance were isolated from blood or urinary tract infections from six hospitals located in Egypt from June 2001 to November 2007 by the Naval Medical Research Unit No. 3 (NAMRU-3), Cairo, Egypt (Table 2). Isolation of these clinical strains was approved by the Institutional Review Board (IRB) at NAMRU-3. As all identifiers were previously stripped from these strains, the described study was deemed exempt from consideration as Human Subjects research by IRBs at both NAMRU-3 and the Naval Research Laboratory (NRL).

**Table 1:** Reference strains used for confirmation of the ARDM content.

Species	Strain (other name)	Description	Reference
*Acinetobacter baumannii*	17978	Wild type, antibiotic-sensitive strain. Chromosome and plasmids sequenced.	[61]
*A. baumannii*	BAA-1710 (AYE)	Multidrug resistant strain. Chromosome and plasmids sequenced.	[55]
*A. baumannii*	AB4857 (MRSN939)	Multidrug resistant strain. Genome sequenced.	[13]
*A. baumannii*	AB5075 (MRSN959)	Multidrug resistant strain. Genome sequenced.	[13]
*A. baumannii*	AB5256 (MRSN961)	Multidrug resistant strain. Genome sequenced.	[13]
*A. baumannii*	AB5711 (MRSN1310)	Multidrug resistant strain. Genome sequenced.	[13]
*Escherichia coli*	25922	Wild type, antibiotic sensitive QC strain for antimicrobial susceptibility testing.	[62]
*Klebsiella pneumoniae*	700603 (K6)	ESBL (SHV-18) containing QC strain for antimicrobial susceptibility testing.	[63]
*K. pneumoniae*	700721 (MGH78578)	Contains multiple ESBLs. Chromosome and plasmids sequenced.	[64]
*K. pneumoniae*	BAA-1705 (D05/07)	Positive control for Modified Hodge Test. Contains KPC carbapenemase.	[65]
*K. pneumoniae*	BAA-2146	Multidrug-resistant strain; produces New Delhi metallo-β-lactamase	[19], [66]
*Staphylococcus aureus*	700699 (Mu50)	Methicillin-resistant *S. aureus*. Genome sequenced.	[67]
*Staphylococcus epidermidis*	35984 (RP62A)	Biofilm producer. Methicillin-resistant strain. Genome sequenced.	[68]
*Enterococcus faecalis*	700802 (V583)	Vancomycin-resistant *Enterococcus*. Chromosome and plasmids sequenced.	[69]

**Table 2:** Clinical isolates from Egyptian hospitals.

Strain	Species	Isolation date	Site^a^
N1	*A. baumannii*	06/05/2001	CUH
N2	*A. baumannii*	06/05/2001	CUH
N3	*A. baumannii*	06/05/2001	CUH
N9	*A. baumannii*	03/05/2006	CUH
N7^b^	*E. coli*	11/04/2007	TBRI
N16	*E. coli*	09/10/2000	AFH
N21	*E. coli*	09/03/2002	AFH
N23	*E. coli*	10/23/2002	ASU
N24	*E. coli*	01/15/2003	SAH
N28	*E. coli*	12/22/2005	ALX
N11	*K. pneumoniae*	06/05/2001	CUH
N19	*K. pneumoniae*	07/31/2001	AFH
N25	*K. pneumoniae*	09/22/2003	ALX
N26	*K. pneumoniae*	09/10/2003	AFH
N29	*K. pneumoniae*	11/29/2006	ALX

a Clinical sites from which the isolates were obtained. CUH – Cairo University Hospital, Cairo, Egypt; AFH – Abbasia Fever Hospital, Cairo, Egypt; ASU – Assiut Fever Hospital, Assiut, Egypt; SAH – Shebeen Al Kom Hospital, Shebeen Al Kom, Egypt; ALX – Alexandria Fever Hospital, Alexandria, Egypt; TBRI – Theodor Bilharz Research Institute, Cairo, Egypt.

b This ESBL strain containing *bla*_CTX-M-15_ has been previously described under the designation of E450 (35).

### Antibiotic susceptibility testing of clinical isolates from Egyptian hospitals

The sensitivity of clinical strains to 20 antibiotics and antibiotic combinations: ampicillin, ampicillin+sulbactam, ticarcillin+clavulanic acid, piperacillin, carbenicillin, cephalothin, cefotaxime, ceftazidime, cefopodoxime, ceftriaxone, cefepime, aztreonam, imipenem, gentamycin, amikacin, tetracycline, chloramphenicol, sulfamethoxazole+trimethoprim, ciprofloxacin and nalidixic acid was determined using disk diffusion assays according to CLSI guidelines [14]. Not all isolates were tested using all of these antibiotics (see Table S1). The extended spectrum β-lactamase (ESBL) phenotypes of all *E. coli* and *K. pneumoniae* isolates were determined using double disk diffusion and the metallo-β-lactamase phenotypes of *A. baumannii* were determined using E-test Metallo-β-Lactamase Strips which contained a combination of imipenem and imipenem/EDTA (BioMerieux, Marcy l'Etoile, France) according to the manufacturer's instructions.

### ARDM

**Figure 1:**
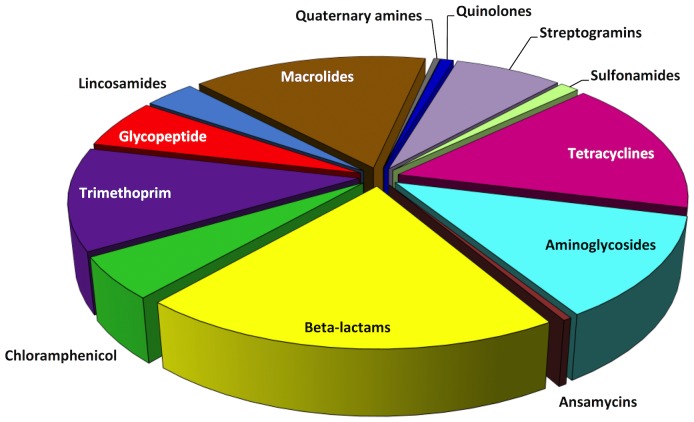
ARDM content allocations for genes conferring resistance to different classes of antimicrobial compounds. Two hundred thirty-nine different resistance determinants are represented by probes on each sub-array.

The ARDM version 1 is a custom designed microarray for use with the ElectraSense platform [15] (CustomArray, Bothell, WA). Electrochemical interrogation of biotinylated amplicon fragments is accomplished using a horseradish peroxidase-streptavidin conjugate with the redox mediator, 3,3′,5,5′-tetramethylbenzidine (TMB). Each 4×2K microarray contains four identical sub-arrays of 2,240 individually addressable microelectrodes functionalized with DNA probe sequences of 30 to 35 nucleotides in length. The probes (Table S2) were derived from 239 genes that confer resistance to quaternary ammonium compounds, streptothricin, and 12 different classes of antimicrobials: aminoglycosides (n = 29), ansamycins (n = 1), β-lactams (n = 48), chloramphenicol (n = 12), diaminopyrimidines (n = 29), glycopeptides (n = 14), lincosamides (n = 8), macrolides (n = 37), quinolones (n = 5), streptogramins (n = 17), sulfonamides (n = 3) and tetracyclines (n = 38) (Figure 1); some determinants (e.g., multidrug efflux pumps) confer resistance to multiple families of antimicrobials. Probe sequences were optimized for uniform melting temperatures and to avoid target cross-hybridization to the extent possible. Each of the 239 genes is represented on the ARDM by a range of 6 to 10 probes, with the most frequent representation comprising four or five pairs of duplicate probes per gene. Chip content includes alleles derived from a wide variety of bacteria: *Firmicutes* (including bacilli, clostridia, lactobacilli; 38% total chip content), *Bacteroidetes* (2% total content), *Actinobacteria* (9% total content), and α-, β-, ε-, and γ-*Proteobacteria* (*Enterobacteriales*, *Aeromonadales*, *Pseudomonadales*, *Vibrionales*, and *Pasteurellales*; 49% total content).

### DNA purification and processing

Genomic DNA was extracted from isolate cultures grown overnight on MacConkey agar using the MagNA Pure LC system (Roche Diagnostics Corp., Indianapolis, IN) or MasterPure DNA and RNA Complete Purification Kit (Epicentre Biotechnologies, Madison, WI) and quantified using the Qubit fluorometer (Invitrogen/Life Technologies, Grand Island, NY) and BR dsDNA QuantIT DNA quantification reagent set (Invitrogen). Ten ng of each DNA sample was subjected to whole genome amplification using the GenomiPhi V2 reagent kit (GE Healthcare, Piscataway, NJ) according to manufacturer's instructions, with the reaction mixture incubated for 90 min at 30°C and subsequently for 10 min at 65°C to stop the reaction. Two µg of the resulting high molecular weight amplicons were fragmented for 1 min at 37°C using Fragmentation Reagent (DNase I, 0.045 units/µg DNA) and Fragmentation Buffer (GeneChip Resequencing Assay Kit, Affymetrix, Sanata Clara, CA) in a total reaction volume of 60 µl. The enzyme was subsequently inactivated by incubation at 95°C for 10 min. The amplified and fragmented DNA was then purified using DNA Clean & Concentrator −5 kit (Zymo Research, Irvine, CA) and labeled using the ULS Platinum Bright Biotin nucleic acid labeling kit (Kreatech Diagnostics, Durham, NC) according to the manufacturer's instructions (total reaction volume of 10 µl). Following purification of the labeled fragments using the kit's Kreapure columns, the samples were hybridized to the ARDM.

### ARDM hybridization and data analysis

Hybridization of the biotinylated amplicon fragments to the ARDMs was performed according to the manufacturer's instructions, except that pre-hybridization and hybridization temperatures were increased to 60°C (from 50°C recommended by the manufacturer). Four samples were analyzed on each 4×2K microarray slide (one sample for each sub-array). Following overnight hybridization, each array was processed using CustomArray's ElectraSense Detection Kit and interrogated on the ElectraSense reader while substituting a streptavidin-peroxidase conjugate (S104PHRP, Fitzgerald Industries, North Acton, MA) for the Biotin Labeling Solution provided, as this highly polymerized conjugate was previously shown to improve detection sensitivity in electrochemically interrogated assays [16]. All data obtained from the ElectraSense reader were analyzed with custom developed Perl scripts using the following criteria for positive/negative signal determinations: individual probes were designated positive when their signal exceeded a threshold defined as the average signal of all microarray probes (excluding the top 5%) plus three standard deviations (SD). Each gene was considered present if 30 or 40% of its probes provided positive signal determinations (depending on the thresholding algorithm used).

### PCR

Clinical isolates from Egyptian hospitals were tested for the presence of alleles from the *bla*_SHV_, *bla*_TEM_, *bla*_CTX-M_ and *bla*_OXA_ families of ESBLs using a multiplex PCR assay [17]. As this assay was not designed to detect *bla*_OXA-9_, an additional PCR was required to detect this gene using the following amplification conditions: 95°C for 2 min, followed by 30 cycles of 95°C for 30 s, 53°C for 30 s, and 72 for 1 min [18]. See Methods S1 for information on methods used for PCR confirmation of tetracycline resistance genes. PCR amplicons were size-confirmed by electrophoresis using 1.2% FlashGel DNA cassettes (Lonza, Walkersville, MD). The identities of the detected amplicons were further verified by DNA sequencing (Eurofins MWG Operon, Huntsville, AL).

## Results and Discussion

The overall efficacy of the ARDM v.1 was first determined using a series of reference strains. Once an appropriate thresholding algorithm was determined for the desired levels of sensitivity and specificity, the 15 clinical isolates obtained from six hospitals in Egypt were subjected to ARDM analysis. As these isolates were originally selected based on their high levels of resistance to β-lactams and/or expression of ESBL phenotypes, results for hybridization to β-lactam/ESBL-specific probes on the ARDM were subsequently confirmed by PCR and DNA sequencing. ARDM detection of tetracycline resistance genes was also confirmed using the same molecular techniques.

### Determination of optimal algorithm(s) for positive determination and confirmation of probed content

**Figure 2:**
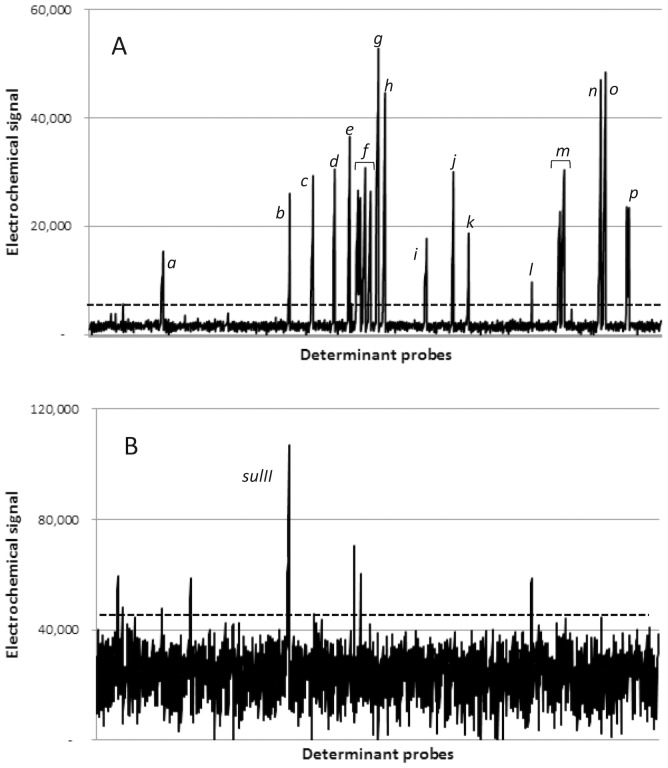
Electrochemical signals from MDR reference strain, *A. baumannii* BAA-1710 (A) and antibiotic sensitive reference strain, *A. baumannii* 17978 (B). Each data point represents one probe; data are sorted according to alleles represented by each probe. In each panel, the horizontal dotted line indicates the “probe threshold” used to determine whether probes were considered positive (mean of lowest 95% probes+3 SD). For panel A, peaks correspond to the following alleles: ***a***: *tet(G)* [9/9 probes positive]; ***b***: *cat4* [5/7 probes positive]; ***c***: *dfrA1* [9/9 probes positive]; ***d***: *catA1* [6/8 probes positive]; ***e***: *arr-3* [8/8 probes positive]; ***f*** *: aph6/str(B)* [8/10 probes positive], *aph3″/str(A)* [10/10 probes positive], *aadA1b* [10/10 probes positive], *aadB* [10/10 probes positive]; ***g***: *qacEΔ1* [10/10 probes positive]; ***h***: *aadA2* [2/9 probes positive - allele deemed negative], *aadA1* [7/7 probes positive]; ***i***: *bla*_VEB_ [9/9 probes positive]; ***j***: *bla*_OXA-10_ [7/7 probes positive]; ***k***: *dfrA10* [8/10 probes positive]; ***l***: *bla*_PSE-1/CARB_ [2/10 probes positive – allele deemed negative]; ***m***: *aacC1* [9/9 probes positive], *aphA1* [10/10 probes positive]; ***n***: *ant(2′)-Ia* [10/10 probes positive]; ***o***: *sulI* [10/10 probes positive]; ***p***: *tet(A)* [9/9 probes positive]. For panel B, the *sulII* allele (peak indicated) was deemed positive with 7/7 probes positive; other alleles had one or two positive probes, but were deemed negative.

Based on results for each of the 2,241 target-specific probes, background signals from microelectrodes with non-hybridized probes could be very clearly distinguished from signals resulting from those with positive probes (Figure 2 and Figure S1). For each microarray, a “probe threshold” was calculated as the mean signal of the 2,138 probes with the lowest signals (95% total probe content on each array) plus three standard deviations. Thus, the probe threshold calculation accounts for differences in overall microarray sensitivity, as well as the variability between negative probes. Once the positive probes were determined for each array, each allele (represented by 6–10 probes) was subjected to another thresholding algorithm, which deemed the allele positive or negative based on the percentage of its probes that were above the “probe threshold.” Fourteen reference strains (Table 1) were used to confirm the efficacy of ARDM probe content and to optimize the threshold algorithm for determination of positive alleles. This panel included MDR strains representing both Gram-negative and Gram-positive bacteria and containing a variety of antibiotic resistance markers. It also included some wild type strains with few or no antibiotic resistance determinants.

#### Initial algorithm testing using commercially prepared DNA

**Figure 3:**
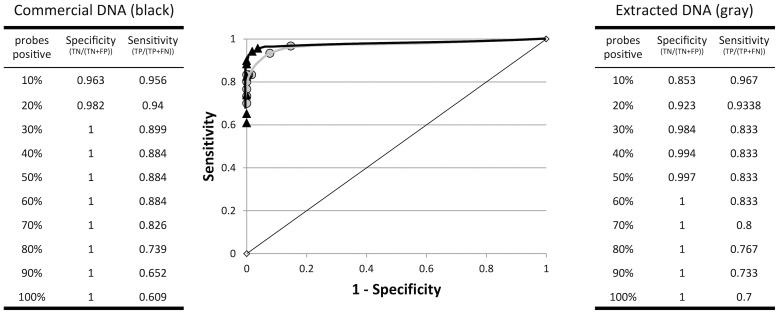
Receiver operating characteristic curves for reference strain data. Specificity and sensitivity data for commercial DNA preparations of sequenced strains (six strains; phenol-chloroform-extracted) are indicated in black. Analogous data for DNA preparations extracted in our laboratories are presented in gray (four strains).

DNA preparations from six strains with sequenced, annotated genomes were initially used to narrow the threshold conditions for positive/negative allele determinations for the desired range of array sensitivity and specificity. Comparing the positive alleles determined under each threshold (10–100% probes positive for each allele) with their presence within the published gene sequences, sensitivity and specificity were calculated and a receiver operating characteristic (ROC) curve was generated (Fig. 3). The desired sensitivity (>80%) and specificity (>99%) were obtained when positive determinations were based on 30–70% of the allele's representative probes meeting “probe threshold” requirements. Under these conditions, no false-positive alleles were detected, and between nine and 13 false-negative alleles were encountered, depending on the stringency of the algorithm.

Several non-sequenced reference strains with confirmed allelic content (*E. coli* 25922, *K. pneumoniae* strains 70603, BAA-1705, BAA-2146) were similarly analyzed using the same set of algorithms. In *E. coli* 25922, an antimicrobial-sensitive strain, only the intrinsic ABC transporter genes, *macA* and *macB*, were detected under any but the least stringent threshold algorithms. In the *bla*_SHV-18_-producing strain, *K. pneumoniae* 700603, 100% of the probes representing *bla*_SHV-1_ and *bla*_SHV-5_ on ARDM were considered positive, confirming the high identity of these sequences and the *bla*_SHV-18_ gene (99% identical). Similarly, a number of alleles from the *bla*_NDM-1_-containing reference strain, *K. pneumoniae* BAA-2146, were also strongly positive, with 100% of their probes deemed above “probe threshold” values (*bla*_FEC_, *bla*_CTX-M-3_, *bla*_SHV-1/PIT-1_, *bla*_LAT-1/CMY_, *aac(6′)-ib*, *aph3″/str(A)*, *aph6/str(B)*, *qacEΔ1*, *sulI*, *sulII*, *dfrA14*), indicating their strong identity to PCR- and sequence-confirmed alleles [19]. Interestingly, however, other alleles on the ARDM that were closely related to this strain's confirmed content were not as strongly positive (e.g., *bla*_CTX-M-12_, *bla*_CTX-M-32_, *bla*_TEM-1_, *bla*_SHV-5_, *aac(6)-ib*, *aadA1b*, *aadA2*, *aadA1*, *mph(A)*, *mph(K)*, *bla*_TEM-10_, *aac(3)-III*, *tet(A)*). These results are likely due to sequence divergence between strains, and illustrate the presence of highly conserved and less conserved sequences among the ARDM content.

The trio of alleles representing the *aad(A1/A2)* family of aminoglycoside adenyltransferases exemplify the importance of having an appropriate threshold algorithm to reflect both conserved and non-conserved sequences. Since *K. pneumoniae* strain BAA-2146 possesses *aadA2*, it was not surprising that 8/9 probes for *aadA2* were positive. However, only 2/7 probes for *aadA1* and 4/10 probes for *aadA1b* were deemed positive, in spite of the 89% sequence identity of *aadA1* and *aadA1b* with *aadA2*. In contrast, *A. baumannii* BAA-1710, which possesses two copies of *aadA1*, had 100% positive probes for both *aadA1* and *aadA1b*, but only 2/9 probes positive for *aadA2*. Thus, each strain showed its appropriate pattern of positive probes from these alleles, but positive determination for the *aadA1*/*aadA2* family of determinants would be highly dependent on which gene is represented on the microarray and the threshold algorithm used. These results also underscore the need for the careful selection of probes for the detection of families of closely related resistance genes. In practice, due to finite space available on the microarrays, tradeoffs must be made between the breadth of the microarray content and the depth of resolution for particular gene families.

#### Algorithm testing with laboratory-extracted DNA

To determine whether the same algorithms for positive allelic determination was appropriate for DNA prepared in our laboratory, a similar ROC analysis was performed with four genome-sequenced *A. baumannii* strains (Figure 3, gray curve). As evident from the ROC curve, the ARDM is a still statistically valid test for correct determination of the covered resistance genotypes, albeit somewhat less accurate for lab-extracted strains than those prepared commercially using phenol-chloroform extraction. This latter set of ROC analyses also assisted us in selecting the thresholding conditions giving the desired specificity and sensitivity for samples prepared in our laboratories. Under the lowest stringency algorithm previously determined to provide acceptable sensitivity and specificity (30% positive probes for each allele), 15 false positives were observed. However, by requiring 40% positive probes for each allele, the number of false-positives was decreased by 60%, while maintaining the specificity and sensitivity desired. We therefore decided to use this algorithm (40% of allele-specific probes positive) for the analyses of unknown clinical isolates. Table 3 shows the results from ARDM analyses for all 14 reference strains tested using the selected threshold algorithm. The efficacy of hybridization for over 50 of the 239 alleles represented on the ARDM was confirmed by PCR, DNA sequencing, or both. Alleles confirmed positive included those from both Gram-negative and Gram–positive species and conferring resistance to β-lactams, aminoglycosides, macrolides, tetracyclines, sulfonamides, chloramphenicol, quaternary amine compounds, glycopeptides, rifampin and lincosamides. Of the sequenced reference strains tested, *A. baumannii* AYE (BAA-1710) and *K. pneumoniae* 700721 each had at least 19 positive alleles with the potential to render these strains resistant to eight and six different classes of antimicrobials, respectively. Of the non-sequenced strains, *K. pneumoniae* BAA-2146 (*bla*_NDM-1_-positive) had 21 positive alleles conferring resistance to six classes of antimicrobials; in addition to PCR confirmation of these alleles [19], antibiograms provided by ATCC further support the phenotypic traits provided by these alleles (http://www.atcc.org/attachments/19037.pdf).

**Table 3:** ARDM analysis of reference strains.

Strain	ARDM hybridization positive^a^	False positive	False-negative
Sequenced reference strains – confirmed content			
*A. baumannii* BAA-1710	*bla*_OXA-10/PSE-2_, *bla*_VEB-1_, *aacC1, aadA1, aadA1b, aadB, ant(2″)-Ia, aphAI, saph3′/str(A), aph6/str(B), tet(A), tet(G), arr-3, catA1, cat4, qacEΔ1, sulI, dfrA1, dfrA10*	*-*	*bla*_OXA-7_, *aadA2, dfrA15, floR*
*A. baumannii* AB4857	*ant(2″)-Ia, qacEΔ1, sulI*	*arr-3, bla*_KPC-1_, *tet(30), vcaM*	*-*
*A. baumannii* AB4857	*aac(6)-Ib, aac(6′)-Ib, aadA1b, aadA2, aadB, ant(2″)-Ia, aph3″/str(A), aph6/str(B), bla*_GES_ *(bla*_GES-1_, *bla*_GES-2_), *dfrA7, qacEΔ1, sulI*	-	-
*A. baumannii* AB5256	*aac(3)-III, aph3″/str(A), aph6/str(B), sulII, tet(B)*	*-*	*-*
*A. baumannii* AB5711	*aacC1, aph3″/str(A), aph6/str(B), sulII*	*tet(30), norA*	*bla*_PER_
*A. baumannii* 17978	*sulII*	*-*	*-*
*E. faecalis* 700802	*erm(B), lsa(A), vanB, vanB2*	-	-
*K. pneumoniae* 700721	*bla*_OXA-9_, *bla*_SHV_ *(bla*_SHV-1/PIT-2_, *bla*_SHV-5/CAZ-4_), *bla*_TEM-1_, *bla*_TEM-10_, *aac(6)-Ib, aac(6′)-Ib, aadA1, aadA1b, aadB, ant(2″)-Ia, aphAI, aph3′/str(A), aph6/str(B), tet(D), catA1, cat4, qacEΔ1, sulI, sulII*	*-*	*bla*_LEN-1_, *aadA2*
*S. aureus* 700699	*mecA, ant(9)-Ia, erm(A), ble, erm(33), norA, tet(38)*	-	*tet(S), tet(M)*
*S. epidermidis* 35984	*blaZ, mecA, aadE, ant(9)-Ia, aphA3, aph(3′)-III, dfrA, erm(A), erm(33), sat4*	***-***	*-*
Reference strains, not sequenced			
*E. coli* 25922	*macA, macB*		
*K. pneumoniae* 70603	***bla*** **_SHV_** ***(bla*** **_SHV-1/PIT-2_, ** ***bla*** **_SHV-5/CAZ-4_)**, *aadB, ant(2″)-Ia, qacEΔ1, sulI*		
*K. pneumoniae* BAA-1705	***bla*** **_KPC-1_, ** ***bla*** **_OXA9_, ** ***bla*** **_SHV_** ***(bla*** **_SHV-1/PIT-2_, ** ***bla*** **_SHV-5/CAZ-4_** ***), bla*** **_TEM-1_**, *aac(6)-Ib, aac(6′)-Ib, aadA1, aadA2, aphAI, mph(A), mph(K), catA1, cat4, qacEΔ1, sulI, dfrA12*		
*K. pneumoniae* BAA-2146	***bla*** **_FEC-1_, ** ***bla*** **_CTX-M-3_, ** ***bla*** **_CTX-M-12_, ** ***bla*** **_CTX-M-32_, ** ***bla*** **_LAT-1/CMY_,** ***bla*** **_SHV_** ***(bla*** **_SHV-1/PIT_, ** ***bla*** **_SHV-5/CAZ_** ***), bla*** **_TEM-1_, ** ***aac(3)-III aac(6)-Ib, aac(6′)-Ib, aadA1b, aadA2, aph3″/str(A), aph6/str(B), mph(A), mph(K), qacEΔ1, sulI, sulII, dfrA14***		***bla*** **_TEM-10_, ** ***tet(A)***

a ARDM-positive genes that have been validated by sequencing or PCR for strains whose genomes have not been fully sequenced are shown in bold.

Several false-negative alleles were observed in our analyses of the reference strains. In all but two cases, the genes represented on the chip (*dfrA15*, *floR*, *aadA2*, *bla*_LEN_, *bla*_PER_, *tet(S)*) were less than 90% identical to the sequence of the reference strains tested, and therefore might not be expected to hybridize to the short probes used in this type of microarray. As a result, probes representing more distantly related alleles will be incorporated into future ARDM versions. Of the two false-negative alleles on the ARDM that were >90% identical to the reference strains, all of the *tet(M)* probes had at least three mismatches and all but two of the *bla*_OXA-7_ probes had at least two mismatches from the reference strain sequences. Given the overall high identity between the sequences used to construct the ARDM and the reference strains, it is clear that redesigned probes will need to have more conserved regions of each gene represented in future versions of the ARDM.

The source for the false-negatives observed with *K. pneumoniae* BAA-2146 is not known, as this strain's genome has not yet been fully sequenced. Therefore, while the presence of a *tet(A)* allele was previously confirmed by PCR, sequence non-homology within the probed regions may be responsible for its poor hybridization on the ARDM. Somewhat more puzzling is the false-negative determination for *bla*_TEM-10_. Although sequences for *bla*_TEM-1_ and *bla*_TEM-10_ are 99% identical, *K. pneumoniae* BAA-2146 hybridized strongly to *bla*_TEM-1_ sequences (8/9 probes positive), but poorly to *bla*_TEM-10_ probes (2/6 probes). While redesign of these probes may improve ARDM performance, representation of two such highly homologous alleles on the ARDM (discussed below) is not required unless a higher degree of redundancy is desired.

Presumptive false-positive results were observed with two of the four *A. baumannii* strains with published sequence contigs. While each of these alleles met the criteria for positive detection (40% probes positive), in all cases a maximum of four probes were deemed positive. Furthermore, several sets of the *tet(30)*, *norA*, and *vcaM* probes gave positive signals in many other samples, including reference strains for which there were no analogous sequences found. The redesign of probes targeting these alleles in subsequent microarray designs (e.g., ARDM v.2) has already begun to address the shortcomings of the first generation ARDM to reduce both false-negative and false-positive identifications.

### Analysis of clinical isolates from Egyptian hospitals

Recent surveillance efforts in the Middle East have indicated that the majority of healthcare-acquired, MDR infections are due to Gram-negative organisms [20]. A panel of *A. baumannii*, *E. coli* and *K. pneumoniae* blood and urine infection isolates (Table 2) was selected for characterization of their underlying genotypes. These three species represent a significant proportion (in some studies, over half) of the Gram-negative organisms associated with nosocomial infections throughout the Middle East [20], [21], [22], [23]. Using the selected threshold algorithm, ARDM analyses revealed the presence of five to 17 determinants (Tables 4 and 5) in each of the clinical isolates. In each case, alleles conferring resistance to at least three classes of antimicrobials were identified.

**Table 4:** ARDM v.1, PCR and phenotypic β-lactamase/ESBL profiles of the clinical and reference strains used in this study.

Clinical strains	Species	β-lactamases/ESBLs detected (ARDM/PCR)					Phenotype					
		SHV	TEM	CTX-M^a^	OXA	OXA9	CAZ	CTX	ATM^b^	IMP	ESBL	MBL
N1	*A. baumannii*	−/−	−/−	−/−	−/−	−/−	R	R	R	R	N/A	+
N2	*A. baumannii*	−/−	+/+	−/−	−/−	−/−	R	R	R	R	N/A	−
N3	*A. baumannii*	−/−	−/−	−/−	−/−	−/−	R	R	R	R	N/A	+
N9	*A. baumannii*	−/−	+/+	−/−	−/−	−/−	R	R	R	R	N/A	+
N7	*E. coli*	−/−	−/−	+(1)/+	+/+	−/−	N/A	N/A	N/A	N/A	+	N/A
N16	*E. coli*	−/−	+/+	+(1)/+	+/+^c^	−/−	R	R	R	S	+	N/A
N21	*E. coli*	−/−	+/+	+(9)/+	−/−	−/−	S	R	I	S	+	N/A
N23	*E. coli*	−/−	+/+	+(9)/+	−/−	−/−	S	R	I	S	+	N/A
N24	*E. coli*	−/−	+/+	+(1,9)/+	+/+^c^	−/−	R	R	R	S	+	N/A
N28	*E. coli*	−/−	+/+	+(1)/+	+/+	−/−	R	R	R	S	+	N/A
N11	*K. pneumoniae*	+/+^c^	+/+	+(9)/+	−/−	−/−	R	R	R	S	+	N/A
N19	*K. pneumoniae*	+/+	+/+	−/−	−/−	+/+	R	I	R	S	+	N/A
N25	*K. pneumoniae*	+/+	+/+	+(1)/+	+/+^c^	+/+	R	R	R	S	+	N/A
N26	*K. pneumoniae*	+/+	+/+	−/−	−/−	+/+	R	R	R	S	+	N/A
N29	*K. pneumoniae*	+/+	+/+	+(1)/+	+/+^c^	+/+	R	R	R	S	+	N/A

N/A – not available. R – resistant, I – intermediate, S – sensitive.

a The identified family to which the CTX-M allele belongs is indicated in parentheses.

b While no official ATM resistance criteria for *A. baumannii* are available, all isolates of this species designated as resistant (R) in the above table showed no zone of inhibition around ATM disks.

c Weakly positive detection by PCR.

**Table 5:** Genetic MDR profiles from clinical isolates, excluding ESBLs.

Strain	Species	Hybridization positive determinants^a^									False positives^c^
		β-lactams^b^	aminoglycosides	macrolides	TET	CHLOR	QAC	SUL	TRI	FQ	
N1	*A. baumannii*		*aadB, ant(2″)-ia, aph3″/str(A)*	*erm(35)*	*tet(39)*						
N2	*A. baumannii*		*aadA1, aadA1b, aadB, aadC1, ant(2″)-Ia, aphA1*		*tet(39), tet(A)*	*catA1, cat4*	*qacEΔ1*	*sulI*			
N3	*A. baumannii*		*aadB, ant(2″)-Ia, aph3″/str(A), sph6/str(B)*		*tet(39)*					*norA*	*tet(38), tet(30), tet(B)*
N9	*A. baumannii*		*aadA1, aadA1b, aadC1, aphA1*	*mef(A)*			*qacEΔ1*	*sulI*			
N7	*E. coli*		*aac(6′)-Ib*	*macA, macB*					*dfrA17*		
N16	*E. coli*		*aac(3)-III, aac(6′)-Ib, aadA1, aadA1b, aph3″/str(A), aph6/str(B)*	*macA, macB, mph(B)*	*tet(A)*	*catA1, cat4*	*qacEΔ1*	*sulI, sulII*	*dfrA1*		
N21	*E. coli*	*ampC/penA*	*aph3″/str(A), aph6/str(B)*	*macA, macB, mph(A), mph(K)*	*tet(B)*	*catA1, cat4*		*sulII*	*dfrA14*		
N23	*E. coli*	*ampC/penA*	*aadA1, aadA1b, aphA1, aph3/str(A), aph6/str(B)*	*macA, macB, mph(A), mph(K)*		*catA1, cat4*	*qacEΔ1*	*sulII*	*dfrA1, dfrA14*		
N24	*E. coli*		*aac(6′)-Ib, aphA1, aph3/str(A), aph6/str(B)*	*macA, macB*	*tet(B)*	*catA1, cat4*		*sulII*			
N28	*E. coli*		*aac(3)-III, aac(6)-Ib*	*macB*	*tet(B)*	*catA1, cat4*	*qacEΔ1*	*sulI*	*dfrA17*		
N11	*K. pneumoniae*		*aadA1b, aadA2* family, *aphA1, aph3/str(A), aph6/str(B)*	*ere(A2)*	*tet(D)*		*qacEΔ1*	*sulI, sulII*	*dfrA19*		
N19	*K. pneumoniae*		*aadA1*				*qacEΔ1*	*sulI*			
N25	*K. pneumoniae*		*aac(3)-III, aac(6)-Ib, aadA1, aadA1b*		*tet(D)*				*dfrA14*		
N26	*K. pneumoniae*		*aac(3)-III, aadA1, aadA1b, aph3/str(A), aph6/str(B)*			*catA1*	*qacEΔ1*	*sulI, sulII*	*dfrA14, dfrA15*		
N29	*K. pneumoniae*		*aac(3)-III, aac(6′)-Ib, aadA1, aadA1b*		*tet(D)*				*dfrA14*		

a Abbreviations: TET – tetracyclines; CHLOR – chloramphenicol; QAC – quaternary ammonium compounds; SUL – sulfonamides; TRI – trimethoprim; FQ - fluoroquinolones.

b Genes responsible for resistance to β-lactams, which are not included in Table 3.

c Based on results obtained with the reference strains and PCR validation (data not shown) and may reflect false positives and/or truncated genes.

### ARDM detection of β-lactam resistance genes and confirmation by PCR and sequencing

A significant proportion of the ARDM v.1 was devoted to genes conferring resistance to β-lactam antibiotics due to their importance in clinical settings. The most important mechanism of β-lactam resistance in Gram-negative bacteria relies on the production of β-lactamases. Due to the rise of broad-spectrum β-lactamases such as ESBLs and carbapenemases among nosocomial isolates in the Middle East [20], [24], we sought to confirm the presence of these genes in the Egyptian isolates by PCR and DNA sequencing to document in detail the prevalence and spread of specific alleles in the analyzed collection.

In parallel with ARDM analyses, DNA from each of the 15 clinical isolates was subjected to multiplex and individual PCR for the *bla*_SHV_, *bla*_TEM_, *bla*_CTX-M_ and *bla*_OXA_ β-lactamase families (Table 4). Multiplex and individual PCRs were performed using the same, previously published primers [17]. In each case where PCR generated the appropriately sized product, the amplicons were purified and sequenced to determine the specific allele present.

The ARDM v.1 content covers genes for *ampC* cephalosporinases and the *bla*_TEM_, *bla*_SHV_, *bla*_OXA_ and *bla*_GES_ families of β-lactamases (some of which are ESBLs and carbapenemases), as well as other families of β-lactamase determinants encoding exclusively ESBLs (e.g., *bla*_ACC_, *bla*_CTX-M_, *bla*_VEB_, *bla*_PER_, and *bla*_LAT-1/CMY_) or carbapenemases (*bla*_KPC_, *bla*_SME_, *bla*_IMP_, *bla*_VIM_, *bla*_SPM_, *bla*_GIM_, and *bla*_SIM_). Although several of the reference strains were ARDM-positive for *bla*_VEB_, *bla*_GES_, and *bla*_LAT-1/CMY_ (in agreement with their published gene contents [25], [26]), only alleles belonging to the *ampC*, *bla*_TEM_, *bla*_SHV_, *bla*_OXA_ and *bla*_CTX-M_ families were detected in the Egyptian isolates. In all of the *Enterobacteriaceae* isolates tested, alleles specific for multiple families were detected; the presence of multiple β-lactamases/ESBLs from different groups has been widely observed in other studies of Egyptian *Enterobacteriaceae* strains [27], [28], [29].

None of the carbapenemases represented on the ARDM v.1 were detected in any of the clinical isolates tested. The absence of these alleles in the *E. coli* and *K. pneumoniae* isolates was supported by their sensitivity to imipenem. In contrast, the *Acinetobacter* isolates were phenotypically resistant to imipenem but were negative for hybridization to any of the carbapenemase probes. It is very likely that the *A. baumannii* isolates harbored one or more carbapenem-hydrolyzing class D β-lactamases, which are commonly associated with *Acinetobacter*. Studies with the four (carbapenem-resistant) *A. baumannii* reference strains obtained from MRSN, for which genome sequences are available, indicate the presence of *bla*_OXA-51_ in all four, and *bla*_OXA-23_ in two (data not shown). As a result, probes for *bla*_OXA-51_, *bla*_OXA-23_ and other carbapenem hydrolyzing OXAs will be incorporated into future versions of the ARDM.

*Bla*_TEM_ was the most frequently detected family of β-lactamase genes by ARDM analyses. All 12 ARDM-positive results were confirmed by PCR, as were *bla*_TEM_-negative results. *Bla*_TEM_ genes were found in two of the *A. baumannii* isolates, in five of the six *E. coli* strains tested, and in all of the *K. pneumoniae* strains tested. Similar distributions of *bla*_TEM_ have been described in several other studies of *Enterobacteriaceae* isolates from Egypt [28], [29], but typically, *bla*_TEM_ is only rarely encountered in *Acinetobacter* [30], [31]. In spite of the presence of two *bla*_TEM_ alleles represented on the ARDM, neither ARDM nor sequence analysis of PCR amplicons was able to distinguish between the broad-spectrum *bla*_TEM_ determinants (*bla*_TEM-1_, *bla*_TEM-2_) and their ESBL derivatives. Although in several cases, sequences of the PCR amplicons could confirm which *family* of *bla*_TEM_ was present (*bla*_TEM-1_ group or *bla*_TEM-2_ group; samples N24 and N25, respectively), an unequivocal identification of a specific allele could not be made for any of the clinical or reference samples tested.

*Bla*_SHV_ β-lactamases were detected in five *K. pneumoniae* strains by ARDM analysis, with all five confirmed by PCR. As with the *bla*_TEM_ alleles, no definitive identification of any specific allele could be made based on the sequence data. At least eight different alleles were identified as equivalent matches for the detected sequences, among them *bla*_SHV-12_ and *bla*_SHV-5_, which have been previously observed in *Klebsiella* from Egypt [29], [32]. No *bla*_SHV_ alleles were detected in the *A. baumannii* or *E. coli* samples tested.

The *bla*_OXA_ family of β-lactamases was represented on the ARDM v.1 by probes specific for four alleles: *bla*_OXA-1_ and *bla*_OXA-7_ (*bla*_OXA-1_ family), *bla*_OXA-9_, and *bla*_OXA-10_. Genes from the *bla*_OXA-1_ group were detected in both *E. coli* and *K. pneumoniae*, but not in *A. baumannii*. In each case, results were confirmed by PCR and DNA sequencing, and it was determined that the allele present in each was *bla*_OXA-1_. *Bla*_OXA-9_ was present in four of the five clinical *K. pneumoniae* isolates and these results were confirmed as well using a *bla*_OXA-9_-specific PCR. Two of these *K. pneumoniae* samples (N25, N29) possessed genes representing both OXA-1 and OXA-9 families. None of the 15 clinical isolates tested was ARDM-positive for genes from the *bla*_OXA-10_ family, which includes ESBLs such as *bla*_OXA-11_, *bla*_OXA-14_, *bla*_OXA-16_ (also known as expanded-spectrum OXAs) and the carbapenem-hydrolyzing class D β-lactamase, *bla*_OXA-128_. While information on the presence and spread of OXA-type enzymes in Egypt is limited to a single case report describing *bla*_OXA-1_ and *bla*_OXA-10_ containing *Pseudomonas aeruginosa* strains [33], our results suggest that both OXA-1 and OXA-9 type enzymes may be common among *E. coli* and *K. pneumoniae* strains isolated from hospital environments in Egypt.

Genes belonging to CTX-M family of ESBLs were detected in the *E. coli* and *K. pneumoniae* isolates, but not in any of the *A. baumannii* samples. *Bla*_CTX-M-1_ group alleles (which include *bla*_CTX-M-3_, *bla*_CTX-M-12_, *bla*_CTX-M-32_, *bla*_FEC-1_) were found in four *E. coli* isolates and in two of the five *K. pneumoniae* isolates. Additionally, alleles representing the *bla*_CTX-M-9_ family (which include *bla*_CTX-M-9_, *bla*_CTX-M-13_) were found in half of the *E. coli* isolates and in one *K. pneumoniae* isolate. One isolate (*E. coli* N24) contained alleles from both *bla*_CTX-M-1_ and *bla*_CTX-M-9_ groups. To our knowledge, this isolate is the first Egyptian strain documented to harbor two *bla*_CTX-M_ genes belonging to distinct families. None of the clinical isolates were positive for genes from the *bla*_CTX-M-2_ and *bla*_CTX-M-8_ groups. All ARDM detection results for CTX-M family genes were confirmed by PCR and DNA sequencing determinations further indicated that the ARDM analyses correctly classified the detected alleles within the appropriate families. In five samples (*E. coli* N7, N16, N24 and *K. pneumoniae* N11, N29), it was possible to identify the individual allele present based on the sequences obtained. In each instance, the gene identified was either *bla*_CTX-M-15_ (*bla*_CTX-M-1_ group) or *bla*_CTX-M-14b_ (*bla*_CTX-M-9_ group). These results agree with other studies documenting the widespread distribution of *bla*_CTX-M-15_ in Egypt [34], [35], [36], and also suggest a high prevalence of *bla*_CTX-M-14_ in the hospitals from which the strains originated. Although *bla*_CTX-M-14_ was previously reported from this region, its prevalence was reported to be relatively low [28], [37], [38], [39].

### ARDM detection of genes conferring resistance to other antibiotic classes

#### Aminoglycoside resistance

ARDM content covers 27 alleles conferring resistance to aminoglycosides, and in all of the clinical isolates tested, at least one aminoglycoside resistance determinant was present. Eight different alleles were detected, representing all three categories of aminoglycoside-modifying enzymes (acetyltransferases, adenylyltransferases, phosphotransferases). Isolates N2, N9, and N16 possessed alleles for all three types of enzymes, with as many as five different alleles detected in the same sample (N16) (Table 5). The most commonly encountered genes were those belonging to the *aadA1/aadA2* family (nine isolates); only one of these isolates harbored an allele more closely homologous to *aadA2* than *aadA1/aadA1b* based on the percentage of probes positive for the three alleles. The *aph3/str(A)* and *aph6/str(B)* genes were co-localized in seven isolates, while a single sample (N1) harbored only *aph3/str(A)*. Acetyltransferase genes *aac(3)-iii* and *aac(6)-ib/aac(6′)-ib* were detected in 50–60% percent of the *K. pneumoniae* and *E. coli* isolates, often together. Similar profiles for *aac(3)-III*, *aac(6)-ib/aac(6′)-ib*, *aph3/str(A)*, *aph6/str(B)*, and *aadA1/aadA1b/aadA2* in isolates of *E. coli* and *K. pneumoniae* from other Cairo hospitals have been described [21], [39]. Two of the *A. baumannii* isolates exhibited strong hybridization to *ant(2″)-Ia/aadB* probes (80–90% positive probes) and two to *aacC1* probes (100% positive probes). However, neither of these alleles was detected in the *E. coli* or *K. pneumoniae* isolates.

#### Macrolide resistance

A number of macrolide resistance determinants were detected in the clinical isolates. Only one of the *K. pneumoniae* isolates (N11) was positive for a resistance determinant, *ere(A2)*, which demonstrated strong hybridization to the ARDM probes. Genes for phosphotransferases *mph(A)* and *mph(K)* – 99% identical in sequence – were also detected in two of the *E. coli* samples (N21, N23), whereas *mph(B)* was identified in *E. coli* N16. The intrinsic ABC macrolide transporter genes, *macA* and *macB*, were detected in all *E. coli* isolates except for sample N28, where only *macB* was detected.

#### Tetracyline resistance

The presence of ARDM-detected tetracycline resistance alleles appeared to be mostly species-specific. *Tet(39)* was detected only in *A. baumannii* (samples N1, N2, N3), *tet(D)* in *K. pneumoniae* (N11, N25, N29) and *tet(B)* mostly in *E. coli* (N21, N24, N28). On the other hand, *tet(A)* was observed in one *A. baumannii* (N2) and one *E. coli* (N16). Individual PCRs were used to confirm these results and revealed that the ARDM-detected *tet(B)*, *tet(38)* and *tet(30)* alleles detected in *A. baumannii* N3 were false-positives. Although the potential for the *tet(30)* and *tet(38)* probes on the ARDM giving rise to false-positive results in *A. baumannii* has been recognized (data not shown), the presence of three false-positive results for this single sample may indicate that contamination occurred during preparation for ARDM analysis; no other confirmed false-positives were encountered during the study of these clinical samples.

Two phenotypically tetracycline resistant samples were found to be negative for all of the 39 tetracycline resistance determinants represented on the ARDM. In one case (*E. coli* N23) the presence of *tet(A)* was confirmed by PCR. This false-negative result was due to unusually high variability in probe signals throughout the array; this high variability increased the “probe threshold” (mean of lowest 95% probes+3 SD), leading to a large number of the *tet(A)* probes erroneously being deemed negative. When analyzed using lower stringency algorithms for calculating the “probe threshold” (e.g., mean of lowest 90% probes+3 SD, mean of lowest 95% probes+2 SD), eight of the nine *tet(A)* probes were deemed positive. The probe signal variability may have also affected detection of the *sulI* allele in this sample (see below).

#### Chloramphenicol resistance

Chloramphenicol resistance is represented by 12 alleles present on the ARDM. Resistance determinants of the *catA1*/*cat4* family were detected in five of the six *E. coli* clinical isolates, as well as in single *K. pneumoniae* and *A. baumannii* isolates. While the presence of these alleles corresponded to phenotypic chloramphenicol resistance, several strains (N3, N9, N11) also demonstrated a resistant phenotype but were negative for any of the determinants represented on the ARDM. This resistance may be due to *catB* or *cmlA* genes (not present on this ARDM version), which have been detected in other *E. coli* isolated from Egyptian hospitals [40]. The *floR* allele, conferring resistance to florfenicol, was not detected in this study.

#### Diaminopyrimidine resistance

Resistance to diaminopyrimidines was represented by probes directed against 29 alleles of dihydrofolate reductase (*dfr*). Although no *dfr* alleles were detected in the *A. baumannii* isolates, five *E. coli* and *K. pneumoniae* isolates were positive for the presence of at least one *dfr* gene; isolates N23 and N26 each harbored two different *dfr* genes. The alleles, *dfrA5*, *dfrA7*, and *dfr12* - observed at high frequency in other studies of MDR isolates from this region [22], [41], [42], [43], [44] – were not found in the clinical strains tested here. However the other *dfr* alleles (*dfrA1*, *dfrA15*, *dfrA17*, and *dfrA19*) detected in our isolates have been well documented by others in various *E. coli*, *K. pneumoniae*, and *Enterobacteriaceae* isolates from this region [22], [41], [42], [43], [44], [45], [46], [47].

Interestingly, the trimethoprim resistance determinant most frequently encountered in the current study was *dfrA14*; to our knowledge, this allele has never been observed in any *Enterobacteriaceae* isolates of Egyptian origin. The high frequency at which it was detected in the current study (33% of isolates) may be indicative of a widely disseminated genetic element within the Cairo area which has not yet been unequivocally identified. Anantham and Hall recently described a plasmid, pCERC1, possessing *dfrA14* within a disrupted *aph3/str(A)* gene and postulated its close relationship to other small, globally distributed plasmids with similar gene sequences of *sulII*→*str(A)*→*dfrA14*→*str(A)*→*str(B)* [48]; several of these potentially related plasmids have been recovered from African isolates [49], [50]. In the present study, two *E. coli* isolates and one *K. pneumoniae* isolate possessing *dfrA14* (N21, N23, N26) also harbored sequences that hybridized strongly to *aph3/str(A)*, *aph6/str(B)*, and *sulII* probes on the ARDM. While we have not ascertained clustering between the four genes detected in these samples, it is possible that sequences homologous to those found in pCERC1 or its related plasmids are present in these Egyptian strains.

#### Determinants directed against other classes of antimicrobials

Resistance to quinolones was represented on the ARDM v.1 by probes directed against *norA* and *qnrA*. Although 12 of the clinical isolates were phenotypically resistant to quinolones, only *A. baumannii* N3 was deemed positive for hybridization to *norA*, and none were positive for *qnrA*. The mechanism for quinolone resistance for the remaining 11 resistant isolates was not determined, but could potentially be due to mutations within the DNA gyrase or DNA topoisomerase IV genes, which would not be detectable using this technology. Six of the clinical isolates that were quinolone-resistant were deemed positive for *aac(6′)-Ib*, which potentially indicated the presence of its quinolone resistance-conferring variant, *aac(6′)-Ib-cr*. However, all six isolates were also resistant to nalidixic acid, indicating the absence of the *aac(6′)-Ib-cr* variant, which is highly specific for quinolones possessing piperazinyl substituents [51]. Alternatively, these isolates may possess other plasmid-borne genes conferring resistance to fluoroquinolones (e.g., *qep*, *qnrB*, *qnrC*, *qnrD*, *qnrS*) not represented on the current version of the ARDM but which will be incorporated into future versions.

Sulfonamide resistance genes were detected in 10 isolates across all analyzed species. *SulI* was found in seven isolates (N2, N9, N11, N16, N19, N26, N28) and was also most likely present in sample N23, which had an unusually high probe signal variability (resulting in false-negative detection of *tet(A)*, see “tetracycline resistance” above). When less stringent criteria (the same as in case of *tet(A)*) were used for “probe threshold” calculations, *sulI* became positive with 7/10 probes. *SulII* was detected in six samples; in three of these samples, it was co-localized with *sulI* (N16, N11, N26). *SulIII* was not detected.

#### Resistance gene clusters

The identification of often-linked groups of characteristic genes allowed us to deduce the likely presence of a number of antimicrobial determinant-harboring genetic structures such as integrons and resistance islands (in addition to the previously mentioned pCERC1 plasmid). In seven of the eight strains where the *qacEΔ1* gene (encoding for resistance to quaternary ammonium compounds) was detected, *sulI* was also present, which may potentially indicate the presence of a class 1 integron. Five of the *qacEΔ1*+/*sulI*+ samples were also positive for the presence of genes from the *aadA1*/*aadA2* family, and two harbored the trimethoprim resistance genes *dfrA1* and *dfrA12*; *aadA1* has been described as the most frequently integrated gene cassette in class 1 integrons [52], [53]. As the ARDM content did not include probes targeting *sat2* or *intI2*, it is unclear whether the coexistence of *aadA1* and *dfrA1* in samples N16 and N23 might also indicate the presence of class 2 integrons (the most widespread of which carries conserved *dfrA1*, *sat2*, and *aadA1* gene cassettes) [54]. The ARDM assay did confirm the presence of resistance island AbaR1 in the *A. baumannii* BAA-1710 reference strain, the strain in which it was first identified [55], as all 16 AbaR1 genes represented on the microarray were hybridization-positive. We also observed specific clusters of resistance determinants in clinical *A. baumannii* isolates N2 and N9 that indicated the potential presence of resistance islands AbaR3 (seven genes: *tet(A)*, *catA1*, *bla*_TEM_, *aphA1b*, *aacC1*, *aadA1*, *sulI*) and AbaR6/R7 (four genes: *aphA1b*, *aacC1*, *aadA1*, *sulI*) [56], respectively.

## Conclusions

Taken together, the results demonstrated that the ARDM is capable of detecting antimicrobial resistance genes and genetic assemblages that are known to confer resistance to first and second line antibiotics that are preferred for the treatment of Gram-negative infections. While the findings of clinical strain analysis were largely in line with previous studies [27], [35], [57], [58], [59], [60], we also detected new alleles (e.g. *dfrA14*, *bla*_OXA-9_) that were clearly circulating in their region of origin and likely missed due to the narrow spectrum of most molecular biology tools. In addition to its strength as a broad screening tool which is complemented by front-end random nucleic acid amplification, the ARDM offers a number of advantages over previously developed tools due to its use of 30–35mer probes and empirically derived detection thresholds (increased specificity), electrochemical hybridization signal detection (increased sensitivity), and a small scanner without any optical systems that can be prone to misalignment in transport (portability). Combined, these attributes make the ARDM a powerful tool for providing molecular epidemiological surveillance information on emerging resistance trends, the identification of resistance gene reservoirs, and potentially providing supporting data to better inform therapeutic decisions in uncharacterized or poorly characterized environments.

## Supporting Information

Methods S1 **Supplemental methods.**
(DOCX)

Figure S1 For top panel (24522), peaks correspond to the following alleles: ***a***: *mac(A)* [5/9] and *mac(B)* [10/10]; ***b***: *bla*_ACC-2_ [1/8] and *mefA* [2/9]; ***c***: *van(C3)* [2/10]; ***d***: *norA* [1/9]; ***e***: *aph(3′)-III* [2/10]; ***f*** *:* bla_PSE-1/CARB-1_ [2/9]; ***g***: bla_CTX-M-2_ [1/10]. All alleles except *mac(A)* and *mac(B)* were deemed negative using the optimized threshold algorithm. For panel (700721), peaks correspond to the following alleles: ***a***: *mecA* [1/10 probes positive; allele deemed negative]; ***b***: *bla*_SHV-1_ [10/10]; ***c***: *bla*_TEM-1_ [9/9] and *bla*_SHV-5_ [6/8]; ***d***: *sulII* [4\6] and *cat4* [3/7]; ***e***: *catA1*[6/8]; ***f***: (cluster) *aph6/str(B)* [6/10], *aph3″/str(A)* [7/9], *aac(6)-Ib* [5/8], and *aad(A1b)* [6/10]; ***g***: *aadB* [4/10], *qacE* ***Δ*** *1* [10/10], and *aad(A1)* [7/7]; ***h***: *aac(6′)-Ib* [3/6]; ***i***: *bla*_OXA-9_ [6/9]; ***j***: *bla*_TEM-10_ [3/6] and *aphA1* [8/10]; ***k***: *sulI* [8/10]; ***l***: *tet(D)* [8/8].
(PDF)

Table S1 **Antibiotic resistance profiles of the clinical isolates^1^.** ^1^ R - resistant, S - sensitive, I - intermediate resistant (according to CLSI criteria). Blank cells - test was not done.
(PDF)

Table S2 **Detailed ARDM content.**
(PDF)
