# Supplementary material for: Molecular Characterization of Multidrug Resistant Hospital Isolates Using the Antimicrobial Resistance Determinant Microarray
Source: PLoS One. 2013 Jul 25;8(7):e69507. doi: 10.1371/journal.pone.0069507 (PMC3723915; doi:10.1371/journal.pone.0069507)
Supplement: Methods S1 — Supplemental methods. (DOCX) [file pone.0069507.s001.docx]

**Molecular Characterization of Multidrug Resistant Hospital Isolates Using the Antimicrobial Resistance Determinant Microarray.**

**Supplemental Methods S1.**

PCR confirmation of *tet* gene detection by ARDM.

The PCR detection of *tet*(A), *tet*(B), *tet*(D), *tet*(30), *tet*(38) and *tet*(39) genes was conducted using primers listed below using previously published thermal cycling conditions.

| **Gene** | **Primer sequences** | **Reference** |
| --- | --- | --- |
| *tet*(A) | GCGCGATCTGGTTCACTCG  AGTCGACAGYRGCGCCGGC | ([1](#_ENREF_1)) |
| *tet*(B) | CGTTTGCTTTCAGGGATCA  ACCATCATGCTATTCCATCC | ([2](#_ENREF_2)) |
| *tet*(D) | GGAATATCTCCCGGAAGCGG  CACATTGGACAGTGCCAGCAG | ([1](#_ENREF_1)) |
| *tet*(30) | CATCTTGGTCGAGGTGACTGG  ACGAGCACCCAGCCGAGC |  |
| *tet*(38) | TTCAGTTTGGTTATAGACAA  CGTAGAAATAAATCCACCTG | ([3](#_ENREF_3)) |
| *tet*(39) | CTCCTTCTCTATTGTGGCTA  CACTAATACCTCTGGACATCA | ([4](#_ENREF_4)) |

**References:**

1. **Aminov, R. I., J. C. Chee-Sanford, N. Garrigues, B. Teferedegne, I. J. Krapac, B. A. White, and R. I. Mackie.** 2002. Development, validation, and application of PCR primers for detection of tetracycline efflux genes of gram-negative bacteria. Appl Environ Microbiol **68:**1786-1793.

2. **Stine, O. C., J. A. Johnson, A. Keefer-Norris, K. L. Perry, J. Tigno, S. Qaiyumi, M. S. Stine, and J. G. Morris, Jr.** 2007. Widespread distribution of tetracycline resistance genes in a confined animal feeding facility. Int J Antimicrob Agents **29:**348-352.

3. **Truong-Bolduc, Q. C., P. M. Dunman, J. Strahilevitz, S. J. Projan, and D. C. Hooper.** 2005. MgrA is a multiple regulator of two new efflux pumps in Staphylococcus aureus. J Bacteriol **187:**2395-2405.

4. **Agersø, Y., and L. Guardabassi.** 2005. Identification of Tet 39, a novel class of tetracycline resistance determinant in Acinetobacter spp. of environmental and clinical origin. J Antimicrob Chemother **55:**566-569.
