# Supplementary material for: Molecular Characterization of Multidrug Resistant Hospital Isolates Using the Antimicrobial Resistance Determinant Microarray
Source: PLoS One. 2013 Jul 25;8(7):e69507. doi: 10.1371/journal.pone.0069507 (PMC3723915; doi:10.1371/journal.pone.0069507)
Supplement: Figure S1 — For top panel (24522), peaks correspond to the following alleles: a: mac(A) [5/9] and mac(B) [10/10]; b: bla ACC-2 [1/8] and mefA [2/9]; c: van(C3) [2/10]; d: norA [1/9]; e: aph(3′)-III [2/10]; f : blaPSE-1/CARB-1 [2/9]; g: blaCTX-M-2 [1/10]. All alleles except mac(A) and mac(B) were deemed negative using the optimized threshold algorithm. For panel (700721), peaks correspond to the following alleles: a: mecA [1/10 probes positive; allele deemed negative]; b: bla SHV-1 [10/10]; c: bla TEM-1 [9/9] and bla SHV-5 [6/8]; d: sulII [4\6] and cat4 [3/7]; e: catA1[6/8]; f: (cluster) aph6/str(B) [6/10], aph3″/str(A) [7/9], aac(6)-Ib [5/8], and aad(A1b) [6/10]; g: aadB [4/10], qacE Δ 1 [10/10], and aad(A1) [7/7]; h: aac(6′)-Ib [3/6]; i: bla OXA-9 [6/9]; j: bla TEM-10 [3/6] and aphA1 [8/10]; k: sulI [8/10]; l: tet(D) [8/8]. (PDF) [file pone.0069507.s002.pdf]

Supplemental Figure S1.

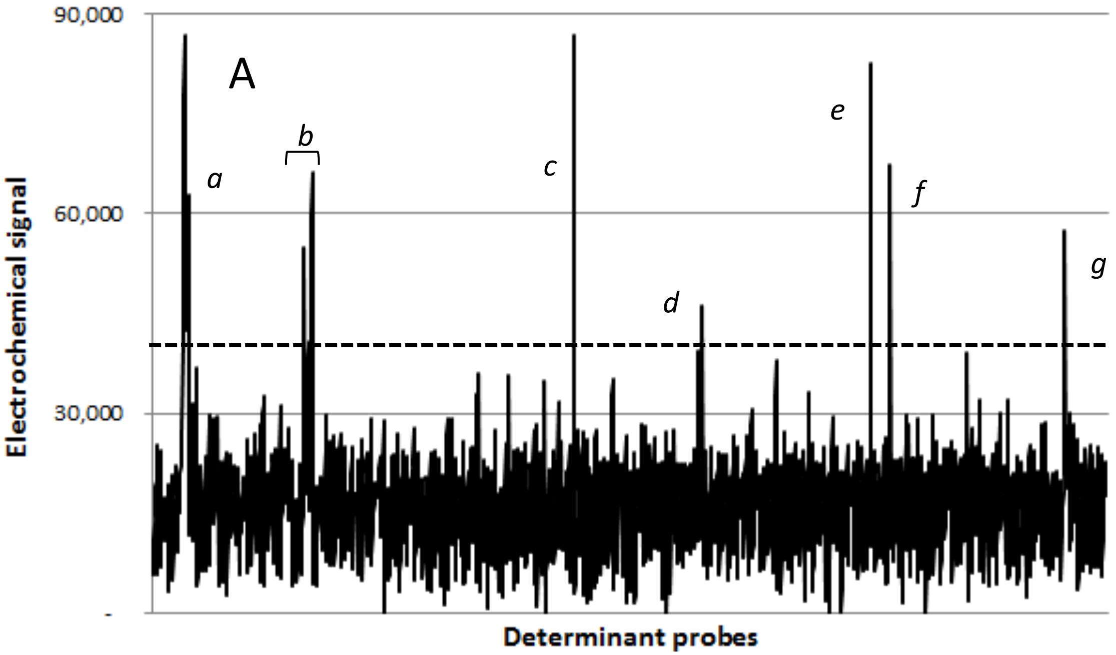

For top panel (24522), peaks correspond to the following alleles: ***a***: *mac(A)* [5/9] and *mac(B)* [10/10]; ***b***: *bla*$_{ACC-2}$ [1/8] and *mefA* [2/9]; ***c***: *van(C3)* [2/10]; ***d***: *norA* [1/9]; ***e***: *aph(3')-III* [2/10]; ***f***: *bla*$_{PSE-1/CARB-1}$ [2/9]; ***g***: *bla*$_{CTX-M-2}$ [1/10]. All alleles except *mac(A)* and *mac(B)* were deemed negative using the optimized threshold algorithm

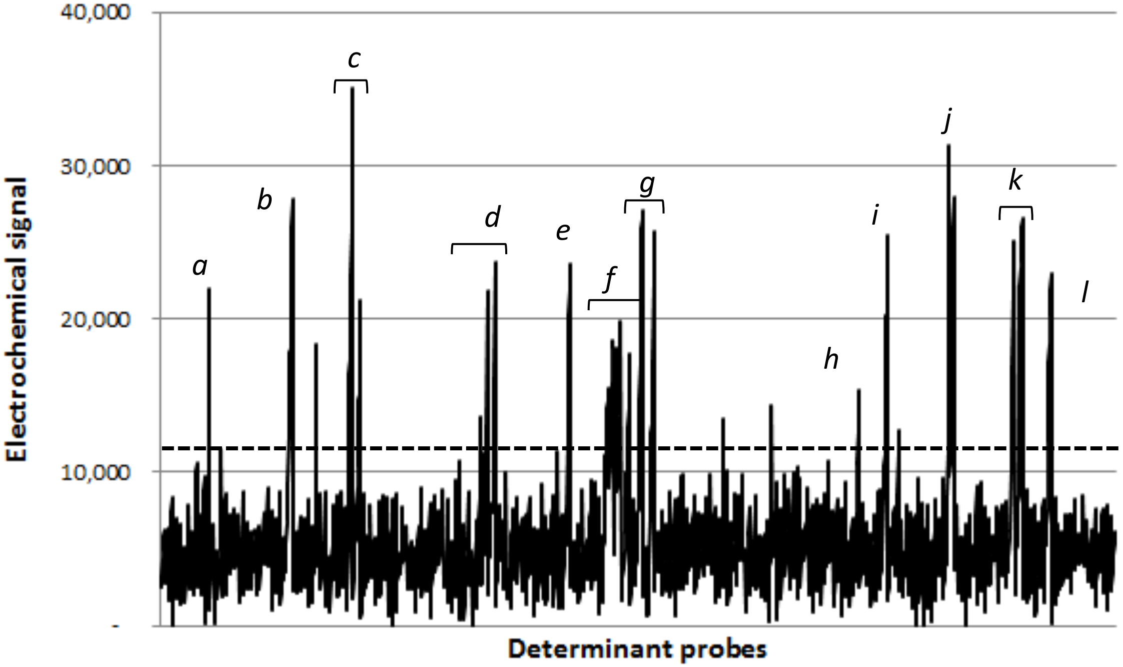

For panel (700721), peaks correspond to the following alleles: ***a***: *mecA* [1/10 probes positive; allele deemed negative]; ***b***: *bla*$_{SHV-1}$ [10/10]; ***c***: *bla*$_{TEM-1}$ [9/9] and *bla*$_{SHV-5}$ [6/8]; ***d***: *sulII* [4\6] and *cat4* [3/7]; ***e***: *catA1*[6/8]; ***f***: (cluster) *aph6/str(B)* [6/10], *aph3"/str(A)* [7/9], *aac(6)-Ib* [5/8], and *aad(A1b)* [6/10]; ***g***: *aadB* [4/10]*, qacEΔ1* [10/10], and *aad(A1)* [7/7]; ***h***: *aac(6')-Ib* [3/6]; ***i***: *bla*$_{OXA-9}$ [6/9]; ***j***: *bla*$_{TEM-10}$ [3/6] and *aphA1* [8/10]; ***k***: *sulI* [8/10]; ***l***: *tet(D)* [8/8]
