# Supplementary material for: Molecular Characterization of Multidrug Resistant Hospital Isolates Using the Antimicrobial Resistance Determinant Microarray
Source: PLoS One. 2013 Jul 25;8(7):e69507. doi: 10.1371/journal.pone.0069507 (PMC3723915; doi:10.1371/journal.pone.0069507)
Supplement: Table S1 — Antibiotic resistance profiles of the clinical isolates1. 1 R - resistant, S - sensitive, I - intermediate resistant (according to CLSI criteria). Blank cells - test was not done. (PDF) [file pone.0069507.s003.pdf]

Supplemental Table S1. Antibiotic resistance profiles of the clinical isolates.[1]

Isolate	Species	AM	SAM	TIM	PIP	CB	CF	CAZ	CTX	CPD	CRO	FEP	ATM	IPM	GM	AN	TE	C	SXT	CIP	NA
N1	*A. baumannii*		R	R	R	R		R	R		R	R	R	R	S	R	I	S	S	R	
N2	*A. baumannii*		R	R	R	R		R	R		R	R	R	R	S	R	I	S	R	R	
N3	*A. baumannii*		I	R	R	R		R	R		R	R	R	R	R	R	R	R	S	R	
N9	*A. baumannii*		R	R	R	R		R	R		R	R	R	R	R	R	R	R	R	I	
N7	*E. coli*																				
N16	*E. coli*	R	I	R			R	R	R	R	R	I	R	S	R	S	R	R	R	R	R
N21	*E. coli*	R	R	I			R	S	R	R	R	S	I	S	S	S	R	R	R	R	R
N23	*E. coli*	R	R	I			R	S	R	R	R	S	I	S	S	S	R	R	R	S	I
N24	*E. coli*	R	R	R			R	R	R	R	R	R	R	S	S	S	R	R	S	R	R
N28	*E. coli*	R	R	R				R	R		R	R	R	S	R	I	R	R	R	R	R
N11	*K. pneumoniae*	R	R	I			R	R	R	R	R	R	R	S	R	R	R	R	R	S	S
N19	*K. pneumoniae*	R	S	I			R	R	I		I	S	R	S	S	R	I	S	S	S	S
N25	*K. pneumoniae*	R	R	R			R	R	R	R	R	R	R	S	R	R	R	S	I	R	R
N26	*K. pneumoniae*	R	R	R			R	R	R	R	R	I	R	S	R	S	R	R	R	R	R
N29	*K. pneumoniae*	R	R	R				R	R		R	R	R	S	R	R	R	S	S	R	R

[1] R - resistant, S - sensitive, I - intermediate resistant (according to CLSI criteria). Blank cells - test was not done.

AM=ampicillin
SAM=ampicillin+sulbactam
TIM=ticarcillin+clavulanic acid
PIP=piperacillin
CB=carbenicillin
CF=cephalotin
CAZ=ceftazidime
CTX=cefotaxime
CPD=cefpodoxime
CRO=ceftriaxone
FEP=cefepime
ATM=aztreonam
IPM=imipenem
GM=gentamycin
AN=amikacin
TE=tetracycline
C=chloramphenicol
SXT=sulfamethoxazole+trimethoprim
CIP=ciprofloxacine
NA=nalidixic acid
