# Supplementary material for: Molecular Characterization of Multidrug Resistant Hospital Isolates Using the Antimicrobial Resistance Determinant Microarray
Source: PLoS One. 2013 Jul 25;8(7):e69507. doi: 10.1371/journal.pone.0069507 (PMC3723915; doi:10.1371/journal.pone.0069507)
Supplement: Table S2 — Detailed ARDM content. (PDF) [file pone.0069507.s004.pdf]

Supplemental Table S2 - detailed ARDM content

Gene	Accesion#:range[1]	Total probes	Unique probes
**β-lactams**			
ampC	AJ237702:4744-5883	9	5
bla1	AF367983:626-1555	10	5
bla2	AF367984:791-1561	9	5
blaACC-2	AF180952:1565-2737	8	5
blaCcrA/CfiA	EU595393:1722-2471	9	5
blaCTX-M-2	X92507:6-881	10	5
blaCTX-M-3	EF570052:240-1115	6	3
blaCTX-M-8	AF189721:274-1149	9	5
blaCTX-M-9	AF174129:6336-7211	10	5
blaCTX-M-12	AF305837:1-876	6	3
blaCTX-M-13	AY870398:243-1076	8	4
blaCTX-M-32	AM420303:2739-3614	10	5
blaDHA/AmpC/MOR-1	Y16410:987-2126	9	5
blaFEC	AB098539:61-936	10	5
blaFOX-1	X77455:701-1849	10	5
blaGES-1	EF219163:1-795	7	4
blaGES-2	AF326355:1-864	6	5
blaGIM-1	AJ620678:1041-1793	9	5
blaIMP-1	EU686386:1137-1877	10	5
blaIMP-2	AJ243491:539-1279	9	5
blaKPC-1	AF297554:131-1012	9	5
blaKPC-3	AF395881:1-882	6	3
blaLAT-1/LAT-4	X78117:142-1287	9	5
blaLEN-1	AY037780:89-949	10	5
blaMOX	D13304:233-1381	10	5
blaOXA-1	J02967:1359-2189	9	5
blaOXA-7	X75562:135-935	6	5
blaOXA-9	M55547:2314-3138	9	5
blaOXA-10/PSE-2	EU708817:2161-2961	7	5
blaOXY-1	Z30177:349-1224	10	5
blaOXY-2	AJ871866:1-873	10	5
blaPER-2	X93314:278-1204	9	5
blaPSE-1/CARB-2	M69058:146-1012	9	5
blaROB-1	AF022114:303-1220	9	5
blaSHV-1/PIT-2	AF148850:6-866	10	5
blaSHV-5/CAZ-4	AF317502:31-812	8	4
blaSIM-1	AY887066:494-1234	9	5
blaSME-2	AF275256:1-885	8	5
blaSPM-1	AY341249:2291-3121	10	5
blaTEM-1	AF309824:119-979	9	5
blaTEM-10/MGH-1	U09188:198-1005	6	3
blaVEB-1	DQ393569:1955-2854	9	5
blaVIM-2	AF369871:553-1353	10	5
blaZ	M60253:142-987	6	3
mecA	AB096217:20340-22346	10	5
pbp1A	AF446215:1-1766	10	5

pbp5	AF375986:1-668	8	5
penA	L02928:119-1060	10	5
**aminoglycosides**			
aac(3)Id	AY458224:707-1183	9	5
aac(3)III	X13542:186-1046	10	5
aac(3)Iva	X01385:244-1029	10	5
aac(6')Ib	M21682:380-985	6	3
aac(6')Ii	L12710:169-717	10	5
aac(6')Im	AF337947:1215-1751	9	5
aac(6)-Ib	AY103455:143-721	8	5
aac(C1)	U04610:670-1203	9	5
aad(A1)	AY339625:10986-11777	7	4
aad(A1b)	AY128093:1-468	10	5
aad(A2)	AY263741:107-898	9	5
aad(A4)	Z50802:1306-2094	10	5
aad(A7)	AY458224:1259-2056	9	5
aad(B)	AY204504:2058-2591	10	5
aad(E)	AF516335:14900-15808	7	5
aad(K)	M26879:90-944	8	5
ant(2'')-Ia	X04555:1149-1829	10	5
ant(4')-Iia	M98270:136-903	10	5
ant(6)-Ia	DQ112355:2075-2533	9	5
ant(9)-Ia	X02588:331-1113	9	5
aph(2')-Ib	AF337947:272-1171	10	5
aph(2')-Ic	U51479:1-1270	9	5
aph(2')-Id	AF016483:131-1036	9	5
aph(3')-III	M36771:293-1084	10	5
aph(3')-Iva	X03364:277-1065	9	5
aph(A2)	X57709:1-795	10	5
aph(A3)	AF516335:16440-17234	10	5
aph(AI)	U13633:6554-7369	10	5
aph3''/str(A)	AY055428:19405-20208	9	5
aph6/str(B)	AY055428:18569-19405	10	5
**macrolides**			
ere(A)	A15069:247-1281	9	5
ere(A2)	AF512546:716-1942	9	5
ere(B)	A15097:383-1642	9	5
erm(30)/pik(R1)	AF079138:1283-2293	10	5
erm(33)	AJ313523:163-894	10	5
erm(34)	AY234334:355-1200	9	5
erm(35)	AF319779:33-833	7	4
erm(36)/erm(ML)	AF462611:186-1031	8	5
erm(41)	EU177504:3053-3469	6	3
erm(A)	X03216:4551-5282	10	5
erm(B)	M11180:714-1451	9	5
erm(C)	M19652:988-1722	6	3
erm(D)	M29832:430-1293	9	5
erm(F)	M14730:241-1041	9	5
erm(H)/car(B)	M16503:244-1143	9	5
erm(Q)	L22689:262-1035	7	4
erm(U)/lmr(B)	AF237667:101-1546	10	5

erm(X)/erm(CD)	M36726:296-1150	10	5
erm(Z)/srm(D)	AM709783:2817-3665	10	5
mac(A)	AB071145:1-1143	9	5
mac(B)	AB071146:1-1947	10	5
mef(A)	AF227520:3254-4471	9	5
mef(E)	AF376746:1372-2589	9	5
mph(A)	D16251:1626-2531	10	5
mph(B)	AB020531:4167-5075	9	5
mph(D)	AB048591:1-840	9	5
mph(K)	U36578:432-1352	10	5
mphC	AM397632:1-900	9	5
mre(A)	U92073:119-1054	9	5
msr(A)	X52085:343-1809	8	5
msr(B)	M81802:94-624	7	4
msr(D)/mel	AF227521:4607-6070	10	5
msr(SA)	AB013298:487-1953	8	5
msrC	AY004350:496-1974	10	5
**tetracyclines**			
tet(30)	AF090987:1130-2314	8	5
tet(31)	AJ250203:1651-2883	8	5
tet(32)	AJ295238:164-2083	10	5
tet(33)	NC_003227:22940-24163	10	5
tet(34)	AB061440:306-770	10	5
tet(35)/eff(J)	AF353562:2213-3322	10	5
tet(36)	AJ514254:2534-4456	9	5
tet(37)	AF540889:1-327	10	5
tet(38)	AY825285:1-1353	9	5
tet(39)	AY743590:749-1936	9	5
tet(A)	X75761:1059-2258	9	5
tet(B)	V00611:61-1266	9	5
tet(C)	Y19114:1-1138	10	5
tet(D)	X65876:1521-2705	8	5
tet(E)	L06940:21-1238	8	5
tet(G)	AF133140:757-1932	9	5
tet(H)	U00792:716-1918	8	5
tet(J)	AF038993:1084-2280	10	5
tet(K)	M16217:305-1684	10	5
tet(L)c	X08034:188-1564	8	5
tet(L)p	M11036:189-1565	10	5
tet(M)	X04388:131-2050	9	5
tet(Q)	X58717:586-2511	9	5
tet(S)	L09756:447-2372	8	5
tet(T)	L42544:478-2433	10	5
tet(U)	U01917:413-730	9	5
tet(V)	AF030344:462-1721	10	5
tet(W)	AJ222769:3687-5606	9	5
tet(X)	M37699:586-1752	9	5
tet(Y)	AF070999:1680-2855	10	5
tet(Z)	AF121000:11880-13034	10	5
tetA(P)	L20800:1063-2325	10	5
tetB(P)	L20800:2309-4267	9	5

**glycopeptides**			
van(A)	M97297:6979-8010	9	5
van(B)	U00456:62-1090	8	4
van(B2)	AF310953:1-1029	10	5
van(C1)	AF162694:1411-2442	9	5
van(C3)	AY033764:26-1078	10	5
van(D4)	AF277571:1262-2293	10	5
van(D5)	AY489045:4010-5041	8	4
van(E)	AF430807:2976-4034	9	5
van(F)	AF098802:1-476	9	5
van(G)	AF253562:3715-4764	10	5
van(H)	AF516335:2787-3755	10	5
van(S)	M97297:4649-5803	10	5
van(X)	AF516335:4785-5393	9	5
van(Y)	AF516335:5821-6366	10	5
**ansamycins**			
arr-3	AY038837:555-1007	8	5
**multidrug efflux**			
cmeB	DQ333454:1831-4950	10	5
cmr	U43535:646-2025	9	5
vcaM	AB073220:250-2109	9	5
vceA	AF012101:562-1749	9	5
vceB	AF012101:1759-3294	10	5
vcmA	AB063193:176-1549	9	5
vcrM	AB073219:282-1619	8	5
**chloraphenicol**			
cat	M35190:309-932	9	5
cat-86	K00544:145-807	9	5
cat(A1)	AP000342:20342-21001	8	4
cat(D)	X15100:91-729	10	5
cat(P)	L02937:4207-4830	9	5
cat(pC194)	NC_002013:1260-1910	6	3
cat(Q)	M55620:459-1118	10	5
cat(S)	X74948:1-492	9	5
cat(TC)	U75299:657-1373	7	4
cat4	AJ401050:889-1548	7	5
cfr	AJ249217:570-1619	10	5
floR	AB114188:11605-12819	8	5
**bleomycin**			
ble	D86934:51206-51610	8	5
**lincosamides**			
lnu(A)/lin(A)	J03947:645-1130	10	5
lnu(B)/lin(B)	AJ238249:127-930	9	5
lnu(D)	EF452177:19-513	9	5
lnu(F)/lin(F)	AJ561197:1321-2142	8	5
lnu(G)/lin(G)	DQ836009:1310-2131	8	4
lnuC	AY928180:1150-1644	9	5
**MLS (macrolides, lincosamides, streptogramins)**			
lsa(A)	AY225127:41-1537	10	5
lsa(B)	AJ579365:4150-5628	10	5
mdt(A)	X92946:10534-11790	10	5

**quinolones**			
norA	D90119:478-1644	9	5
qnr	AY259086:8075-8731	9	5
**quarternary ammonium compounds**			
qacED1	AY259086:4692-5039	10	5
**streptogramins**			
vat(A)	L07778:258-917	7	5
vat(B)	U19459:67-705	8	5
vat(C)	AF015628:1307-1945	10	5
vat(D)/sat(A)	L12033:162-791	10	5
vat(E)/sat(G)	AF139725:63-707	9	5
vat(E3)	AY008284:1-645	8	4
vat(E5)	AY043209:1-645	7	4
vat(E6)	AY043210:1-645	8	5
vat(E8)	AY043213:1-645	6	4
vat(F)/sat	AF170730:70-735	9	5
vga(A)lc	DQ823382:1-1569	9	5
vga(B)	U82085:629-2287	9	5
vgb(A)/vgh	M20129:641-1540	9	5
vgb(B)	AF015628:399-1286	10	5
**streptothricins**			
sat4	AF516335:15805-16347	9	5
**sulfonamides**			
sulI	X12870:2595-3434	10	5
sulII	AJ313522:449-1264	7	5
sulIII	AJ459418:2979-3770	8	5
**thrimetoprim**			
dfr(A)	AF051916:2823-3308	8	5
dfr(D)	Z50141:82-582	7	4
dfrA1	AJ628353:2654-3112	9	5
dfrA2a	U36276:717-953	9	5
dfrA2d	AJ429132:69-305	6	3
dfrA3	NC_006526:317899-318396	10	5
dfrA3b	AY162283:5616-6095	10	5
dfrA5	AJ419169:117-590	10	5
dfrA6	Z86002:336-809	10	5
dfrA7	AJ419170:118-687	9	5
dfrA8	NC_010064:711-1220	9	5
dfrA9	X57730:726-1259	9	5
dfrA10	L06418:5494-6057	10	5
dfrA12	Z21672:310-807	8	5
dfrA13	Z50802:718-1215	8	5
dfrA14	AJ313522:1873-2346	9	5
dfrA15	DQ989302:248-721	10	5
dfrA16	EU158182:117-590	9	5
dfrA17	AF169041:162-635	8	5
dfrA18	AY034138:7413-7967	10	5
dfrA19	AJ310778:7004-7573	9	5
dfrA20	AJ605332:1304-1813	9	5
dfrA21	AY552589:1-498	10	5
dfrA22	AJ628423:325-822	9	5

dfrA23	AJ968952:245-742	6	3
dfrA24	AJ972619:83-640	9	5
dfrA25	AB280920:132-590	9	5
dfrA26	AM403715:303-854	10	5
dfrA27	EU678897:1039-1512	10	5
Total number of alleles: **276**		**2136**	**1157**

[1] This column contains GenBank accesion number and the range of the sequence used to design microarray probes
